# The In-Situ Observation of Grain Rotation and Microstructure Evolution Induced by Electromigration in Sn-3.0Ag-0.5Cu Solder Joints

**DOI:** 10.3390/ma13235497

**Published:** 2020-12-02

**Authors:** Xing Fu, Min Liu, KeXin Xu, Si Chen, YiJun Shi, ZhiWei Fu, Yun Huang, HongTao Chen, RuoHe Yao

**Affiliations:** 1 School of Electronics and Information, South China University of Technology, Guangzhou 510640, China; fuxing@ceprei.com; 2 Department of Reliability Design Research, China Science and Technology on Reliability Physics and Application of Electronic Component Laboratory, Guangzhou 510610, China; 19s155065@stu.hit.edu.cn (K.X.); chensi@ceprei.com (S.C.); shiyijun@ceprei.com (Y.S.); fuzhiwei@ceprei.com (Z.F.); huangyun@ceprei.com (Y.H.); 3 Department of Materials Science and Engineering, Harbin Institute of Technology, Shenzhen 518055, China

**Keywords:** electromigration, in-situ EBSD, grain rotation, Sn hillock, grain boundary

## Abstract

The in-situ observation of Sn-3.0Ag-0.5Cu solder joints under electromigration was conducted to investigate the microstructure and grain orientation evolution. It was observed that there was a grain rotation phenomenon during current stressing by in-situ electron backscattered diffraction (EBSD). The rotation angle was calculated, which indicated that the grain reorientation led to the decrease of the resistance of solder joints. On the other hand, the orientation of β-Sn played a critical role in determining the migration of Cu atoms in solder joints under current stressing migration. When the angle between the electron flow direction and the c-axis of Sn (defined as *α*) was close to 0°, massive Cu_6_Sn_5_ intermetallic compounds were observed in the solder bulk; however, when *α* was close to 90°, the migration of the intermetallic compound (IMC) was blocked but many Sn hillocks grew in the anode. Moreover, the low angle boundaries were the fast diffusion channel of Cu atoms while the high grain boundaries in the range of 55°–65° were not favorable to the fast diffusion of Cu atoms.

## 1. Introduction

The trend of miniaturization and the integration of electronic devices leads to the reduction of the size of solder joints [1,2]. Accordingly, the current density in the solder joints will increase dramatically [3]. As the current density increases to the threshold value of 1 × 10^4^ A/cm^2^, it will cause a serious electromigration (EM) issue [4,5,6]. Massive atoms migrate from cathode to anode, which causes metallization dissolution or voids formation at the cathode side and the abnormal growth of the intermetallic compounds (IMCs) at the anode side [7,8,9,10,11]. A few studies have reported the effect of temperature and current density on EM; the higher temperature or current density, the faster the failure could be in the solder joints [12,13]. On the other hand, for the most widely used Sn-based solder, the effect of the Sn grain orientation cannot be ignored. β-Sn shows a significant anisotropy due to the body centered tetragonal (BCT) structure (a = b = 0.5831 nm, c = 0.3182 nm). For instance, the diffusion coefficient of Cu atoms along the c-axis of Sn is 2 × 10^−6^ cm^2^/s at 25 °C, which is 500 times faster than that along the a-axis and b-axis and the electrical resistivities of the c-axis and a or b-axis are 13.25 µΩ·cm and 20.27 µΩ·cm, respectively [14]. Therefore, the anisotropy of Sn has a great influence on electromigration damage [15].

Recently, a growing number of scholars have focused on the effects of grain orientation on the EM in solder joints. Lin [16] found that the Sn grain orientation played a vital role in the formation of Cu_6_Sn_5_ IMCs during EM. Chen [17] reported the “polarity effect” and that the interfacial IMCs at the anode side grew rapidly as the c-axis of Sn were parallel to the electron flow direction while no “polarity effect” occurred as the c-axis was perpendicular to that. Han [18] reported the effects of Sn grain on the growth direction of IMCs during EM and found that IMCs tended to grow along the direction of the c-axis. The Sn grain orientation also affected the dissolution of the IMC near the interface. Lee [19] reported that grain orientations played a critical role in dictating the dissolution behavior at the cathode side. Huang [20] also confirmed that the serrated cathode dissolution behavior was closely related to Sn grain orientations. Tasooji [21] found that the effects of grain boundaries on the diffusion behavior of the atoms were more significant than the effects of grain orientations; however, it is not clear whether the grain boundaries played a critical role in determining the diffusion behaviors of solute atoms. On the other hand, EM also induces grain orientation evolution. Harris [22] studied the behavior of grain reorientations in the films of gold and proposed that the rotation rate strongly depended on the grain size. Lloyd [23] reported that the decrease of resistivity was the driving force of grain rotation during EM, which meant that some grains with higher resistivity would self-align to those with a lower resistivity. Shen [24] investigated the evolution of grain orientation during EM by synchrotron microdiffraction and the results indicated that the grain rotation occurred only in the current crowding region and the grain rotation led to a little decrease of resistivity. Chen [25] found that grain orientations at the neck region of the Sn-3.0Ag-0.5Cu (SAC305) solder joints tended to be identical after EM, which was due to the grain rotation and the grains merged in the direction of decreasing resistivity.

However, most of the studies were not based on in-situ observation, which would degrade the accuracy and continuity of the observation. In this work, the in-situ EBSD and in-situ scanning electron microscope (SEM) were conducted to investigate the microstructure evolution during electromigration. The effects of the β-Sn orientation and the grain boundaries on the IMC’s migration and Sn hillock growth, as well as the grain rotation behavior, are reported in this study.

## 2. Materials and Methods

**Figure 1:**
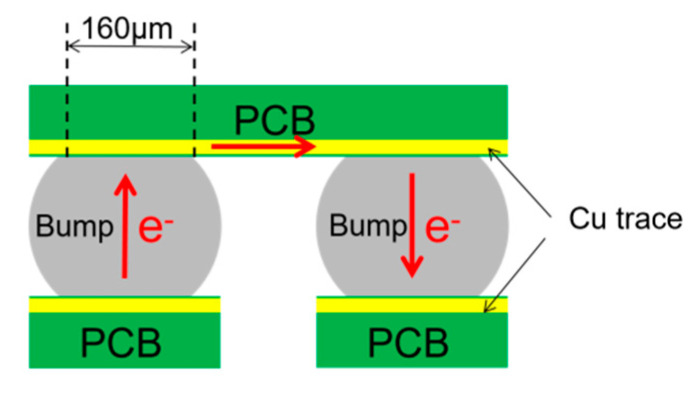
The schematic diagram of the electromigration test for Sn3.5Ag0.5Cu solder joints.

Figure 1 shows the schematic diagram of the electromigration test. The Sn-3.0Ag-0.5Cu (Sn content was 96.5 wt.%, the Ag content was 3.0 wt.% and the Cu content was 0.5 wt.%) solder balls were used in the experiment. The diameters of the solder balls and the Cu pads were 200 μm and 160 μm, respectively. The interconnects between the solder balls and printed circuit boards were prepared by reflowing at 260 °C for 100 s. The prepared samples were mounted in epoxy resin, ground and polished using standard metallographical methods. Ion milling was conducted at 5 kV and 2 mA for different times to further improve the surface quality for EBSD observation. In order to conduct the in-situ SEM and EBSD observation, the surface of the solder joints were kept away from oxidation and contamination during EM. Hence, the sample was placed in a cryogenic probe station (CRX-6.5 K), which could provide a clean high vacuum environment (50 Pa). The samples were applied with a current of 1.6 A (current density: 1.6 × 10^4^ A/cm^2^) for different times and the samples were removed from the probe station every 100 h for SEM and EBSD observation. The SEM was used to investigate the microstructure changes during current stressing. The grain orientation data were collected by an EBSD detector installed on the SEM. Channel 5 software was adopted for the analysis of the scanned data.

## 3. Results and Discussion

### 3.1. Grain Rotation Induced by Electromigration

**Figure 2:**
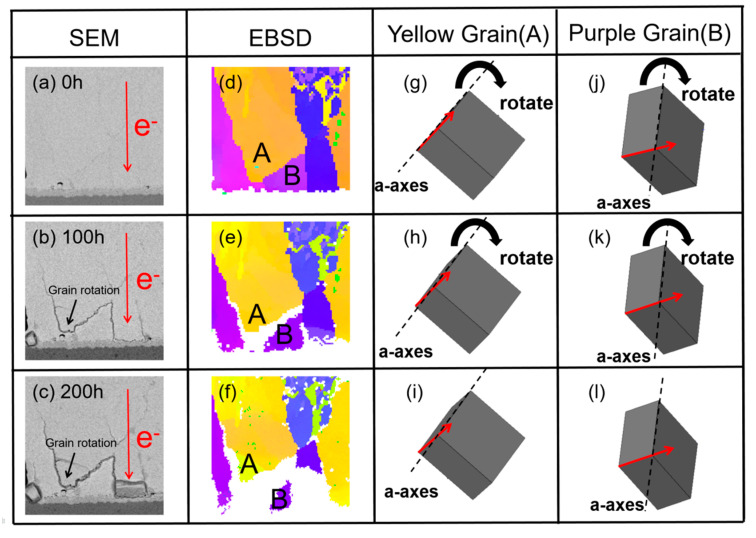
The cross-section of a grain rotation area that was stressed with a current density of 1.6 × 10^4^ A/cm^2^ at 25 °C for different times: (**a**) SEM image for 0 h; (**b**) SEM image for 100 h; (**c**) SEM image for 200 h; (**d**) corresponding electron backscattered diffraction (EBSD) image of (**a**); (**e**) corresponding EBSD image of (**b**); (**f**) corresponding EBSD image of (**c**); (**g**–**i**) the unit cells of yellow grain correspond to (**d**–**f**); (**j**–**l**) the unit cells of purple grain correspond to (**d**–**f**).

Figure 2a–c shows the SEM images of a solder joint under current stressing at different times and the red arrows indicate the electron flow directions. It was clearly observed that grain rotation occurred at the region near the anode side after electrical current stressing for 100 h. The grain rotation phenomenon became more obvious after 200 h. Figure 2d–f shows the corresponding grain orientation maps of the solder joints before and after electromigration for 100 h and 200 h, respectively. The different colors represent different orientations of each grain. It was found that the grain rotation occurred both in the yellow grains and purple grains. The unit cell structures of the yellow grains and purple grains are shown in Figure 2g–i and Figure 2j–l, respectively. After comparing with the unit cell structures at different times, it was noted that the unit cell rotated around the a-axis.

**Figure 3:**
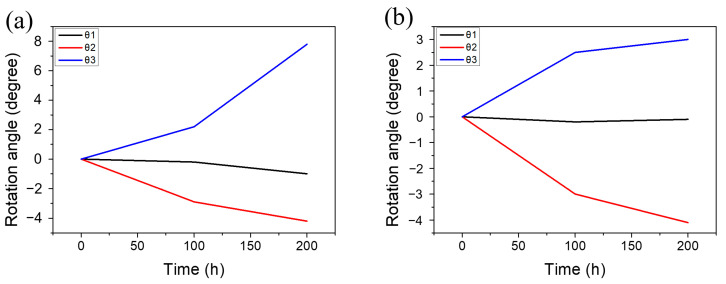
The angle evolution of (**a**) the yellow grain and (**b**) the purple grain. θ1, θ2 and θ3 represent the angle between the current flow direction and the a-, b- and c-axes, respectively.

As β-Sn has a BCT structure, the properties of the a-axis and b-axis were equivalent but different with the c-axis. θ1, θ2 and θ3 represent the angle between the electron flow direction and the a-, b- and c-axis, respectively. Figure 3a shows the angle evolution of the yellow grains. It can be observed that the angle of θ3 increased 7.8° and the angle of θ2 decreased 4.2° after EM for 200 h; however, the angle of θ1 between the a-axis and the electron flow direction did not show a significant change. This indicated that the grain might rotate around the a-axis (long axis). Figure 3b shows that the similar phenomenon was also observed in the purple grains. Therefore, we inferred that the grains tended to rotate around the a-axis.

**Figure 4:**
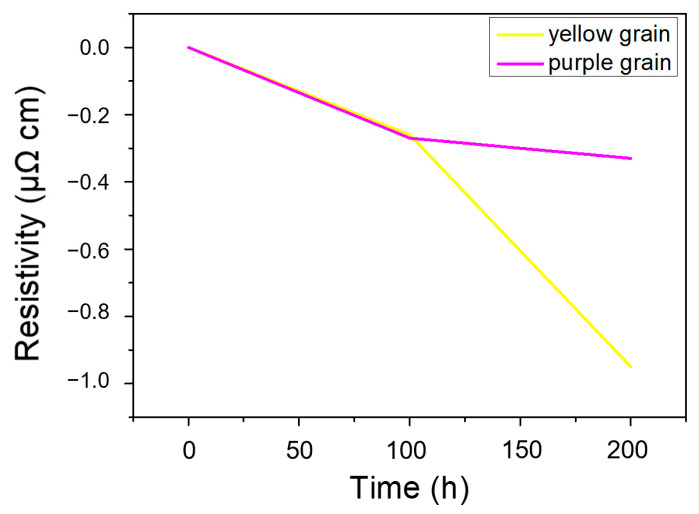
The resistivity evolution of the yellow grain and the purple grain.

Due to the anisotropic nature of Sn, the grain orientation had a significant effect on the resistivity. The resistivity was 13.25 µΩ·cm along the a-axes and b-axes while it was 20.27 µΩ·cm along the c-axis. The resistivity evolution of the yellow grains and purple grains can be calculated based on Equation (1) [26].

(1) $$\sigma =\sigma 1{cos}^{2}\theta 1+\sigma 2{cos}^{2}\theta 2+\sigma 3{cos}^{2}\theta 3$$

where σ is the resistivity of a grain along the given current direction, σ1, σ2 and σ3 represent the resistivities along the a-, b- and c-axis, respectively, and θ1, θ2 and θ3 represent the angles between the current direction and the a-, b- and c-axis, respectively. The results of the resistivity are shown in Figure 4. The results indicated that the grain tended to rotate to reduce the resistance during EM and the grain direction with high electrical conductivity realigned with the current flow. Shen [24] investigated the grain rotation behaviors during EM by in-situ synchrotron and their results also showed that the resistance of the rotated grains decreased slightly.

The mechanism of grain rotation can be explained by the anisotropy of the Sn grain [27]. The grain orientations in the neighboring grains were different, which caused the different flux of vacancy with them. The results in the gradient of vacancy concentrated between the cathode side and the anode side, which corresponded to the stress gradient and finally led to the grain rotation. As shown in Figure 2d, the orientation of the rotated grain (yellow grain) was significantly different from the orientation of the adjacent grains (purple grain). Due to the anisotropic nature of β-Sn, the fluxes of vacancy were different in the two grains, leading to different vacancy concentrations between them.

### 3.2. The Influence of β-Sn Grain Orientation and Grain Boundary on the IMC Migration during EM

**Figure 5:**
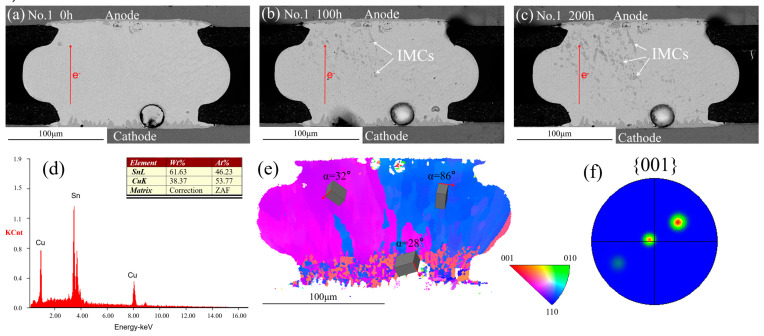
SEM images of the No.1 solder joint after different current stressing times: (**a**) 0 h; (**b**) 100 h; (**c**) 200 h; (**d**) the EDS results of IMCs; (**e**) EBSD map of the No.1 solder joint after reflow; (**f**) pole figure of {001} plane.

**Figure 6:**
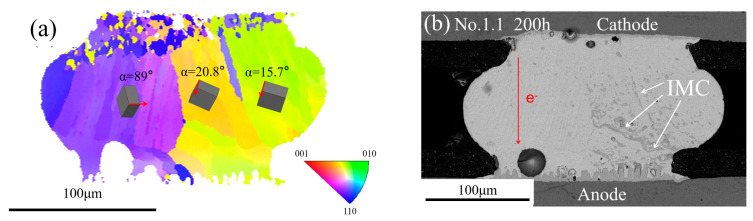
(**a**) EBSD map of the No.2 solder joint after reflow; (**b**) SEM image of the No.2 solder joint after current stressing for 200 h.

Figure 5a–c shows the SEM images of the solder joint before and after EM. The electron flowed from bottom to top. Figure 5a shows the initial morphology; the scallop IMCs were formed at the top and the bottom side after reflow. The composition of the IMCs was identified as Cu_6_Sn_5_ with the help of EDS, as shown in Figure 5d. The SEM images of the No.1 solder joint after current stressing with an upward electron flow of 1.6 × 10^4^ A/cm^2^ for 100 h and 200 h are shown in Figure 5b and Figure 5c, respectively. A mass of Cu_6_Sn_5_ IMC occurred at the left side of the solder joint after 100 h and the Cu_6_Sn_5_ IMC on the left side continued to accumulate with the current stressing times. However, no Cu_6_Sn_5_ IMC was found at the right side during the whole EM process. Figure 5e shows the EBSD map of the No.1 as-fabricated solder joint and the different colors represent different grains. The three colors indicated that the solder joint had three β-Sn grains. Figure 5f shows the {001} pole figure and the three clusters also indicated that there were three β-Sn grains. The unit cells are shown in the EBSD map and the *α* was defined as the angle between the c-axis of Sn and the electron flow. It was found that the Cu_6_Sn_5_ IMC appeared only in small *α* angle grains. A similar phenomenon occurred in the No.2 solder joint, as shown in Figure 6. After 200 h current stressing, no IMC was formed within the large *α* angle grain (89°) while massive IMCs appeared on the small *α* angle grains (15.7° and 20.8°).

**Figure 7:**
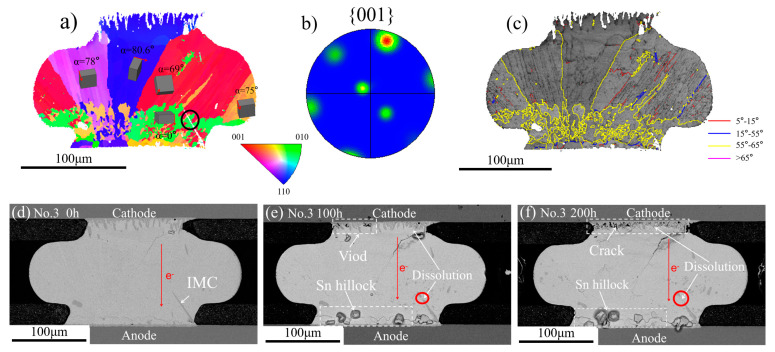
(**a**) EBSD map of the No.3 solder joint after reflow; (**b**) pole figure of {001} plane; (**c**) the grain boundary distribution map; SEM images of the No.3 solder joint after different current stressing times: (**d**) 0 h; (**e**) 100 h; (**f**) 200 h.

The grain orientations of the No.3 solder joint are shown in Figure 7. Figure 7a shows the EBSD map and there were five grains within the solder joints. Figure 7e,f show the microstructure of the No.3 solder joint under the current stressing for 100 h and 200 h, respectively. It can be noted that the Cu_6_Sn_5_ IMC localized in the cathode side was dissolved under the electronic wind, as shown in Figure 7f, which was a normal phenomenon during EM. Another thing worth noting was that the Cu_6_Sn_5_ IMC localized in the anode side was dissolved as shown in Figure 7e,f and a rod-like Cu_6_Sn_5_ IMC localized in the green grains (*α* = 0°) was dissolved while the rest of the parts in the red grains (*α* = 69°) had no significant changes. This was due to the large *α* angle of the red grains near the cathode side, which was not favorable to the diffusion of Cu atoms. In addition, some voids formed between the solder matrix and the IMC layer near the cathode side after 100 h current stressing and the voids evolved into the crack after 200 h, as shown in the Figure 7f. Many Sn hillocks grew in the anode and the growth of Sn hillocks meant that large internal stresses were near the anode side.

The orientations of the Sn grain were related to the IMC’s migration and the Sn hillock growth. The flux of Cu and Sn atoms during EM can be expressed as Equations (2) and (3), respectively [17]:

(2) $$J_{Cu}=\frac{C_{Cu}}{kT}Z_{Cu}^{*}eE\left(D_{a}^{Cu}+D_{c}^{Cu}{cos}^{2}\alpha \right)$$

(3) $$J_{Sn}=\frac{C_{Sn}}{kT}Z_{Sn}^{*}eE\left(D_{a}^{Sn}+D_{c}^{Sn}{cos}^{2}\alpha \right)$$

where $C_{Cu}$ and $C_{Sn}$ are the concentration of Cu and Sn atoms in the per unit volume, respectively, T is the absolute temperature, k is the Boltzmann’s constant, $D_{a}^{Cu}$ and $D_{a}^{Sn}$ are the diffusivity of Cu and Sn atoms along the a-axis of Sn, respectively, $D_{c}^{Cu}$ and $D_{c}^{Sn}$ are the diffusivity of Cu and Sn atoms along the c-axis of Sn, respectively, $Z_{Cu}^{*}$ and $Z_{Sn}^{*}$ are the effective charge numbers of Cu and Sn, respectively, e is the electronic charge and E is the electrical field strength.

From Equation (2), it can be seen that the value of $J_{Cu}$ was proportional to the cos^2^*α*, which indicated that the flux of Cu atoms decreased with the increase of *α*. This was the reason why no IMC appeared within the blue grains (*α* = 86°) of the No.1 solder joint and the purple grains (*α* = 89°) of the No.2 solder joint.

To find out the reason for the Sn growth, the flux ratio of $J_{Cu}$ and $J_{Sn}$ (defined as *λ*) can be expressed as follows [28]:

(4) $$\lambda =\frac{J_{Cu}}{J_{Sn}}=\frac{\left(D_{Cu}^{a}+D_{Cu}^{c}{cos}^{2}\alpha \right)•C_{Cu}Z_{Cu}^{*}}{\left(D_{Sn}^{a}+D_{Sn}^{c}{cos}^{2}\alpha \right)•C_{Sn}Z_{Sn}^{*}}.$$

According to a previous study, $Z_{Cu}^{*}$ is 2 and $Z_{Sn}^{*}$ is 18 [28]. The solubility of Cu in Sn is $1.0\times 10^{-4}.at\%$ and the $D_{Cu}^{a}$ and $D_{Sn}^{a}$ can be expressed as [29]:

(5) $$D_{Cu}^{a}=2.4\times 10^{-3}exp\left(\frac{-33.1\left(\mathrm{kJ}/\mathrm{mol}\right)}{kt}\right)\left({\mathrm{cm}}^{2}/s\right)$$

(6) $$D_{Sn}^{a}=21\;exp\left(\frac{-108.5\left(\mathrm{kJ}/\mathrm{mol}\right)}{kt}\right)\left({\mathrm{cm}}^{2}/s\right)$$

where t is the temperature. Through this calculation, it could be obtained that when the *α* was 0°, *λ* was approximately equal to 800 and when the *α* was 90°, *λ* was equal to 3.2. Hence, the diffusion flux of Cu and Sn was comparable as the *α* closed to 90°. Massive Sn atoms migrated from the cathode to the anode, which left many vacancies near the cathode side and developed into voids and finally a crack. On the other hand, the Sn atoms diffused from the cathode to the anode, leading to the oversaturation of the Sn atoms near the anode side, which caused compressive stress resulting in the growth of the Sn hillocks.

**Figure 8:**
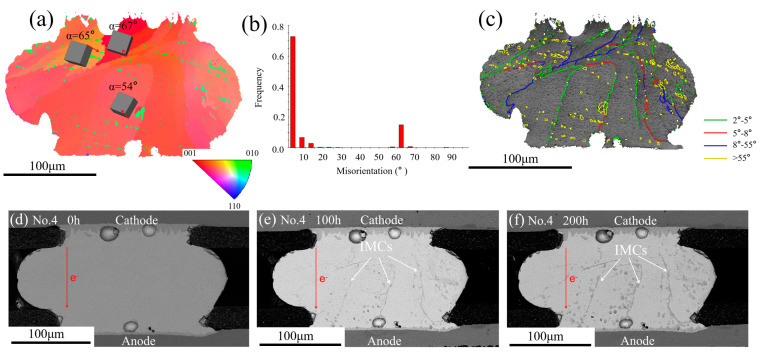
(**a**) EBSD map of the No.4 solder joint after reflow; (**b**) the misorientation distribution map; (**c**) the grain boundary distribution map; SEM images of the No.4 solder joint after different current stressing times: (**d**) 0 h; (**e**) 100 h; (**f**) 200 h.

**Figure 9:**
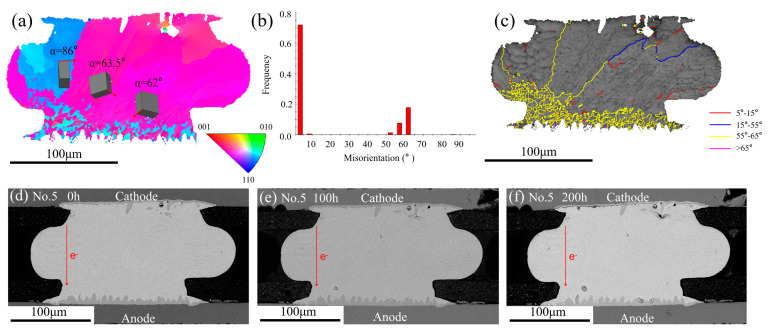
(**a**) EBSD map of the No.5 solder joint after reflow; (**b**) the misorientation distribution map; (**c**) the grain boundary distribution map; SEM images of the No.5 solder joint after different current stressing times: (**d**) 0 h; (**e**) 100 h; (**f**) 200 h.

The grain boundary type also affected the IMC’s migration. Figure 8a shows the EBSD map. The solder joint mainly consisted of one grain with the *α* angle range from 54°to 67°, which was not favorable for the fast diffusion of Cu atoms. Figure 8b shows the misorientation distribution map. It can be seen that most of the misorientation of the grain boundaries was in the range of 0°–15° and 55°–65°. Figure 8c shows the grain boundary distribution map; the green line represented the misorientation from 2° to 5° and the red line represented the misorientation from 5° to 8°. Therefore, most of grain boundaries were low angle grain boundaries. The No.4 solder joint microstructural evolution during the EM test from 0 h to 200 h are shown in Figure 8d–f. A massive Cu_6_Sn_5_ IMC was observed in the solder matrix and it could be noted that the IMCs mainly formed at the low grain boundaries (2°–8°). There were fast diffusion channels of Cu atoms due to a mass of defects in the grain boundaries. As a consequence, the grain boundary diffusion was at least three magnitudes higher than the lattice diffusion along any direction within the grain [30]. However, the accumulation of IMCs was not observed in the No.3 and No.5 solder joints, which contained many high angle grain boundaries. The No.3 solder joint contained many grain boundaries in the range of 55°–65°, as shown in Figure 7c, while no IMCs were observed along the grain boundaries. The same phenomenon occurred in the No.5 solder joint. Figure 9d–f shows the microstructure of the No.5 solder joint at different EM times. Figure 9a shows the EBSD map and the *α* angle of the grains within the bump was larger than 62°. According to the previous analysis, the large *α* angle grain would inhibit the fast diffusion of Cu atoms, hence no obvious IMCs appeared with grains. Figure 9c shows the grain boundary distribution map; it was noted that most of the grain boundaries were labeled with a yellow line (55°–65°) and Figure 9b also confirmed that. Whereas no obvious IMC was found along the high grain boundaries in the No.5 solder joint. In general, the diffusion of Cu atoms along the high angle grain boundaries were faster than along the low angle grain boundaries. However, by comparing the phenomenon of the No.4 and No.5 solder joints, it was interesting to find that both of them contained large *α* angle grains, which were not favorable to the fast diffusion of Cu atoms. In the No.4 solder joint, a massive Cu_6_Sn_5_ IMC appeared along the low grain boundaries whose orientation were just in the range of 2°–8° while no Cu_6_Sn_5_ IMC was found along the high grain boundaries (55°–65°) in the No.5 solder joint. This phenomenon suggested that the type of grain boundaries had significant effects on the diffusion of Cu atoms. The cyclic-twin boundaries defined as the misorientation of grain boundaries were 57.2° and 62.8° and the percentage of the cyclic-twin boundaries with a solder joint went as high as 85% [31,32]. The cyclic-twin boundary was a coherent boundary, which meant fewer defects and low energy. Thus, the Cu atoms were difficult to diffuse along the cyclic-twin grain boundaries. That was why no obvious IMC migrated along the high grain boundaries in the No.5 solder joint.

## 4. Conclusions

In this work, the microstructure evolution of Sn3.0Ag0.5Cu solder joints under current stressing was investigated. Through in-situ EBSD, grain rotation was observed during current stressing and it was found that the Sn grain mainly rotated around the long axis of Sn. In addition, it was revealed that the resistance of the grain decreased with the EM time. As different grain orientations resulted in a different flux of vacancy, the grain rotation strongly depended on the grain orientation of β-Sn. On the other hand, it was found that the Cu_6_Sn_5_ IMC migration under the current stressing had a close relationship with the orientation of β-Sn and a massive Cu_6_Sn_5_ IMC accumulated within the β-Sn grain when the *α*-angle was close to 0°; no obvious Cu_6_Sn_5_ IMC migrated within the β-Sn grain when the *α*-angle was close to 90°. It was also found that the types of grain boundaries had a significant effect on the diffusion of Cu atoms. The low angle boundaries with misorientation in the range of 2°–8° contributed to the diffusion of Cu atoms; however, the high angle boundaries with misorientation in the range of 55°–65° were not favorable to the diffusion of Cu atoms because the high angle boundaries (55°–65°) were cyclic-twin boundaries, which were coherent boundaries with few defects and low energy.
